# Environmental cadmium exposure and the risk of kidney stones: a systematic review and dose-response meta-analysis

**DOI:** 10.3389/fmed.2025.1555028

**Published:** 2025-07-30

**Authors:** Zheng-Ju Ren, Qin Zhang, Na-Xian Tang, Ya-Dong Li, Dong-Liang Lu, An-Long Lin, Chuan Yang, Feng Wang

**Affiliations:** ^1^Department of Urology, The Second Affiliated Hospital of Chongqing Medical University, Chongqing, China; ^2^Department of Radiology, Chongqing Hospital of Traditional Chinese Medicine, Chongqing, China; ^3^Department of Urology, Shenzhen University General Hospital, Shenzhen, China

**Keywords:** kidney stones, cadmium exposure, heavy metal, systematic review, meta-analysis

## Abstract

**Background:**

Recent studies have investigated the relationship between cadmium exposure and kidney stones. Nevertheless, the results remain controversial. Therefore, we performed a comprehensive systematic review and meta-analysis based on the latest evidence to address gaps in the research.

**Methods:**

Medline, Embase, and the Web of Science databases were searched to identify relevant studies up until 31 July 2024. Characteristics and outcomes of the included studies were extracted following the Preferred Reporting Items for Systematic Reviews and Meta-Analyses (PRISMA) guidelines and the Meta-analyses of Observational Studies in Epidemiology (MOOSE) guidelines. A random effects model was used to determine the association between cadmium exposure and the risk of kidney stones.

**Results:**

A total of 17 studies involving 159,011 individuals were included in the meta-analysis. When comparing the highest versus lowest cadmium exposure levels, the overall relative risk (RR) for kidney stones was 1.19 [95% confidence interval (CI): 1.10–1.29]. Subgroup analysis showed that urinary (RR = 1.19; 95%CI: 1.08–1.30) and blood (RR = 1.49; 95% CI: 1.10–2.02) cadmium levels were associated with an increased risk of kidney stones. In contrast to non-cadmium-contaminated areas, both blood (RR = 1.08; 95% CI: 1.00–1.15) and urinary (RR = 1.16; 95% CI: 1.05–1.27) cadmium levels were associated with an increased risk of kidney stones in cadmium-contaminated areas. In the dose–response meta-analysis, we observed a consistent linear positive association between cadmium exposure and the risk of kidney stones. The overall RR for every 1.0 μg/L increase in urinary cadmium levels was 1.07 (95% CI: 1.01–1.13).

**Conclusion:**

Our findings suggest that cadmium exposure is associated with the risk of kidney stones. These findings reinforce the importance of environmental cadmium exposure as a risk factor for kidney stones, extending beyond the influence of conventional risk factors. Efforts to reduce cadmium exposure in the population may help reduce the individual, economic, and societal burdens associated with kidney stones.

**Systematic review registration:**

https://www.crd.york.ac.uk/PROSPERO/myprospero.

## Introduction

Kidney stones are a common condition worldwide, with incidence and prevalence increasing among both children and adults (1–3). Approximately 10 ~ 12% of men and 5 ~ 6% of women are affected by kidney stones (4). Kidney stone formation is a complex process resulting from an imbalance in urine between promoters and inhibitors of crystal formation (5, 6). Various risk factors, such as geography, diet, genetics, and occupation, can affect this balance, resulting in the development of kidney stones (7). Among these risk factors, environmental factors are recognized as important contributors to kidney stone formation (8, 9).

Cadmium, a heavy metal, is one of the most toxic industrial and environmental pollutants, which poses a severe threat to human health (10, 11). Cadmium can enter the body through air, water, soil, and food, and it largely accumulates in the kidneys, liver, bones, and other organs, causing irreversible damage to the target organs (12–14). Previous studies have evaluated different types of heavy metals and their concentrations in urinary stones (15, 16). Notably, significantly higher concentrations of 17 elements, including cadmium, were found in all types of stones (15, 16). In addition, cadmium concentrations were higher in calcium phosphate stones, along with other elements (15). Previous studies have reported that cadmium accumulation causes cellular toxicity and damages multiple organs (17). Long-term exposure to cadmium has been linked to a higher calcium excretion rate and tubular impairment with a loss of reabsorptive capacity, which increase the risk of kidney stone formation (18, 19).

Several observational studies have attempted to address the association between cadmium exposure and the risk of kidney stones. However, their results remain controversial. To better understand this issue, we conducted a comprehensive systematic review and meta-analysis of published literature that investigated the correlation between cadmium exposure and the risk of kidney stones.

## Methods

### Literature search and eligibility criteria

**Figure 1:**
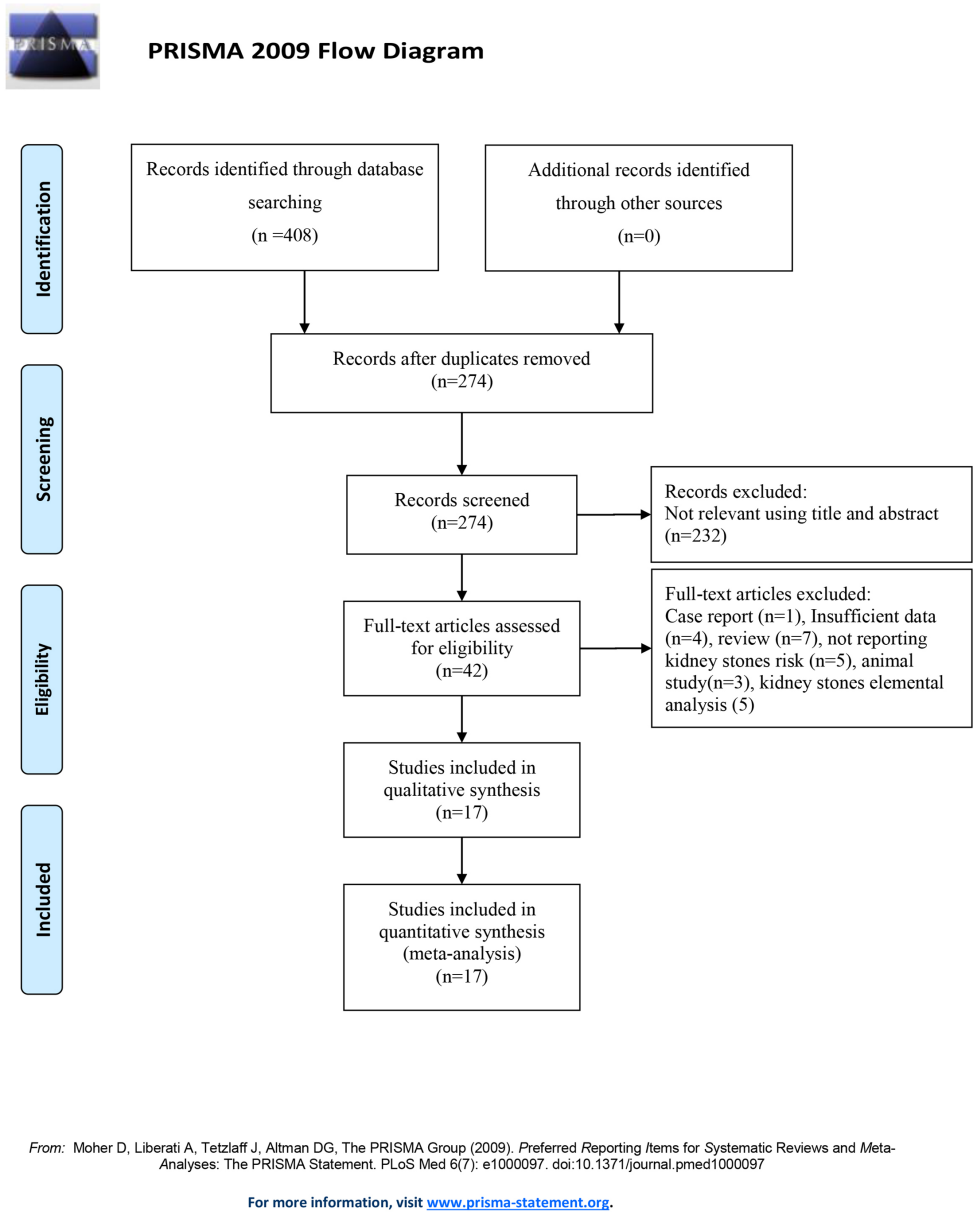
PRISMA flow diagram illustrating the selection of the included studies.

Medline, Embase, and the Web of Science databases were searched up until 31 July 2024. The search terms included “metal exposure OR cadmium” and “kidney stones OR nephrolithiasis OR urolithiasis OR renal stones.” The reference lists of relevant studies were reviewed to identify additional studies. Figure 1 shows the search strategy. Studies were considered eligible if they (1) were published in the English language; (2) had the full text available; (3) evaluated the relationship between cadmium exposure and the risk of kidney stones; (4) provided risk estimates with confidence intervals (CIs) or offered data to calculate these associations; and (5) were case–control, cohort, or cross-sectional studies. The protocol was registered with PROSPERO (CRD42024564167) on 11 July 2024.

### Data extraction

Data were independently extracted by two investigators using a standardized collection form. The relevant data extracted included the following: first author, publication date, study design, study region, sample size, effect estimates [OR, relative risk (RR), HR, or IRR] with 95% CIs, and the potential confounders used for adjustment. Any discrepancies in the results were resolved through discussion with a third investigator.

### Quality assessment

Two investigators used the Newcastle–Ottawa scale (NOS) to conduct quality assessments of case–control and cohort studies (20). A maximum of 9 stars are awarded to each study based on three aspects: 4 stars for the selection of participants, 2 stars for the comparability of groups, and 3 stars for the assessments of outcomes. Scores of 7–9, 4–6, and 0–3 were categorized as high, moderate, and low quality, respectively, for each study. The quality assessment of the cross-sectional studies was conducted following the guidelines provided by the Agency for Healthcare Research and Quality (21). A total of 11 items were included in this self-rating scale, with each item worth one point. The following score categories were used to assess article quality: 0–3 indicated low quality, 4–7 indicated moderate quality, and 8–11 indicated high quality.

### Statistical analysis

The primary outcome was the relative risk of kidney stone incidence. Subgroup analyses of the primary outcome were conducted based on sex and whether the area was cadmium-contaminated. For each study, the risk ratio for kidney stones with the corresponding 95% CI was calculated. A random effects model was used to compute the pooled risk ratio. A chi-squared-based Q test and the I^2^ statistic were performed to evaluate the heterogeneity between studies. If I^2^ is greater than 50% and the *p*-value is less than 0.10, heterogeneity was considered statistically significant. A *Z*-test was performed to assess the significance of the overall RR, and a *p-*value of < 0.05 was considered statistically significant. We carried out a dose–response meta-analysis of the risk of kidney stones according to the methods proposed by Orisini et al. (22) and Berlin et al. (23). Each category’s mean concentration of cadmium was taken as the corresponding dose. When the upper boundary of the highest category was open-ended, the midpoint was calculated by multiplying the lower boundary by 1.5. We set the lowest category to zero, if it was unavailable. The linear and non-linear models were evaluated based on the null hypothesis, with the spline coefficients set to zero. A sensitivity analysis was conducted to evaluate the stability of the results by excluding one study at a time. Potential publication bias was tested using funnel plots, Begg’s test, and Egger’s test. All statistical analyses were conducted using Stata software (version 14.0) (Stata Corporation, College Station, Texas, United States).

## Results

### Search results and study characteristics

The systematic search of articles published up to 31 July 2024 identified 408 articles. After screening the titles and abstracts, we obtained 42 studies for a full-text review. After the full-text review, we finally included 17 published studies comprising 159,011 individuals in the analysis (19, 24–39) (Figure 1). Among these, 1 study was a cohort study, 3 were case–control studies, and 13 were cross-sectional studies. Six of these studies were conducted in America, five in Europe, and six in Asia. Furthermore, 14 studies reported cadmium concentrations measured in urine, 5 studies reported cadmium concentrations measured in blood, and 1 study reported cadmium concentrations measured in dietary sources. The articles were published between 1985 and 2024. The detailed characteristics of all included studies are shown in Table 1. The majority of studies were of medium to high quality. One cross-sectional study was of low quality (Table 1).

**Table 1:** Study population and exposure characteristics of the studies included in the meta-analyses.

Author (year)	Country	Study design	Study quality	Sample size	Biological sample type	Measure of effect	RR (kidney stone risk) (95% CI)	Adjustment factors
Lu et al. (2024) (39)	United States	Cross-sectional study	High	8,515	Urine	OR	1.663 (1.277, 2.167)	Age, sex, ethnicity, education levels, marital status, BMI, hypertension, diabetes, vigorous recreational activities, moderate recreational activities, blood urea nitrogen, creatinine, uric acid, eGFR, and urine creatinine
Ye et al. (2023) (36)	United States	Cross-sectional study	High	9,056	Urine	OR	1.51 (1.10, 2.06)	Sex, age, ethnicity, education, alcohol consumption, smoking, family income/poverty, diabetes, BMI, physical activity, cardiovascular disease, and urinary creatinine
Wang et al. (2023) (38)	United States	Cross-sectional study	Moderate	1,244	Urine	OR	1.87 (0.80, 4.34)	–
Zhao et al. (2023) (24)	United States	Cross-sectional study	Moderate	7,809	Urine	OR	1.85 (1.35, 2.53)	Sex, age, ethnicity, education, household poverty-to-income ratio, marital status, serum cotinine, BMI, urinary creatinine, vitamin C, kidney failure, gout, cancer, activity
Li et al. (2022) (32)	China	Case-control	High	740	Blood	OR	1.61 (1.1, 2.34)	Age, sex, BMI, nationality, marital status, occupation, education level, systolic blood pressure, diastolic blood pressure, smoking status, and drinking status, creatinine, urea, and uric acid
Liu et al. (2022) (25)	China	Cross-sectional study	Moderate	5,792	Urine	OR	0.52 (0.36, 0.75)	Age, BMI, systolic blood pressure, diastolic blood pressure, serum creatinine, glomerular filtration rate, serum urea, uric acid, urine protein, smoking status, and alcohol intake
Huang et al. (2021) (29)	China	Case-control	Moderate	1,572	Urine	OR	Male: 1.41 (0.67, 2.98), Female: 1.69 (0.97, 2.93)	Length of residency, BMI, nationality, family income, education level, smoking, alcohol drinking, and hypertension
Sun et al. (2019) (37)	United States	Cross-sectional study	High	29,199	Urine, blood	OR	Blood^*^: 1.36 (0.98, 1.87), Urine^&^: 2.37 (1.12, 5.04)	Age, sex, ethnicity, body mass index, socioeconomic characteristics (including educational level, marital status, and annual family income), smoking, physical activity, total energy intake, and intakes of calcium, phosphate, sodium, potassium, magnesium, total fluid, alcohol, caffeine, vitamins B6, C, and D, and estimated glomerular filtration rate
Hara et al. (2016) (30)	Belgium	Cross-sectional study	High	1,302	Urine, blood	HR	Blood^*^: 1.13 (0.93, 1.38), Urine^&^: 1.23 (0.98, 1.54)	Sex, age, serum magnesium, and 24-h urinary volume and calcium
Kaewnate et al. (2012) (19)	Thailand	Case-control	High	1,085	Urine	OR	2.73 (1.16, 6.42)	Sex, age, smoking status, and alcohol consumption
Swaddiwudhipong et al. (2011) (26)	Thailand	Cross-sectional study	Moderate	6,748	Urine	OR	Male: 1.093 (1.051, 1.138), Female: 1.039 (0.995, 1.084)	Age, alcohol consumption, body mass index, diabetes, hypertension, and urinary cadmium
Ferraro et al. (2011) (28)	United States	Cross-sectional study	Moderate	15,690	Urine	OR	1.40 (1.06, 1.86)	Age, ethnicity, body mass index, smoking, region of residence, and daily intake of calcium and sodium
Swaddiwudhipong et al. (2010) (33)	Thailand	Cross-sectional study	Moderate	795	Urine	OR	0.99 (0.94, 1.03)	Age, sex, smoking, body mass index, urinary cadmium, diabetes, urinary stone, hypertension, and urinary calcium
Järup et al. (1997) (31)	Sweden	Cross-sectional study	Moderate	46	Blood	OR	5.6 (1.03, 30.2)	–
Järup et al. (1993) (34)	Sweden	Cross-sectional study	Moderate	765	Urine, blood	IRR	Blood^*^: 3.2 (1.3, 8.3) Urine^&^: 1.6 (0.5, 5.0)	–
Elinder et al. (1985) (27)	Sweden	Cross-sectional study	Low	58	Urine	OR	8.9 (1.01, 77.9)	–
Thomas et al. (2013) (35)	Sweden	Cohort	Moderate	68,595	Dietary	HR	Male: 0.97 (0.77, 1.23), Female: 0.99 (0.89, 1.43)	BMI, overweight, obesity, alcohol consumption, cigarette use, and dietary intake of calcium, iron, magnesium, potassium, vitamin B6, and vitamin C

*Blood cadmium levels and kidney stone risk; ^&^Urinary cadmium levels and kidney stone risk; OR, Odds ratio; RR, Relative risk; HR, Hazard ratio; IRR, Incidence rate ratios; BMI, Body mass index; eGFR, Estimated glomerular filtration rate.

### Quantitative synthesis

**Figure 2:**
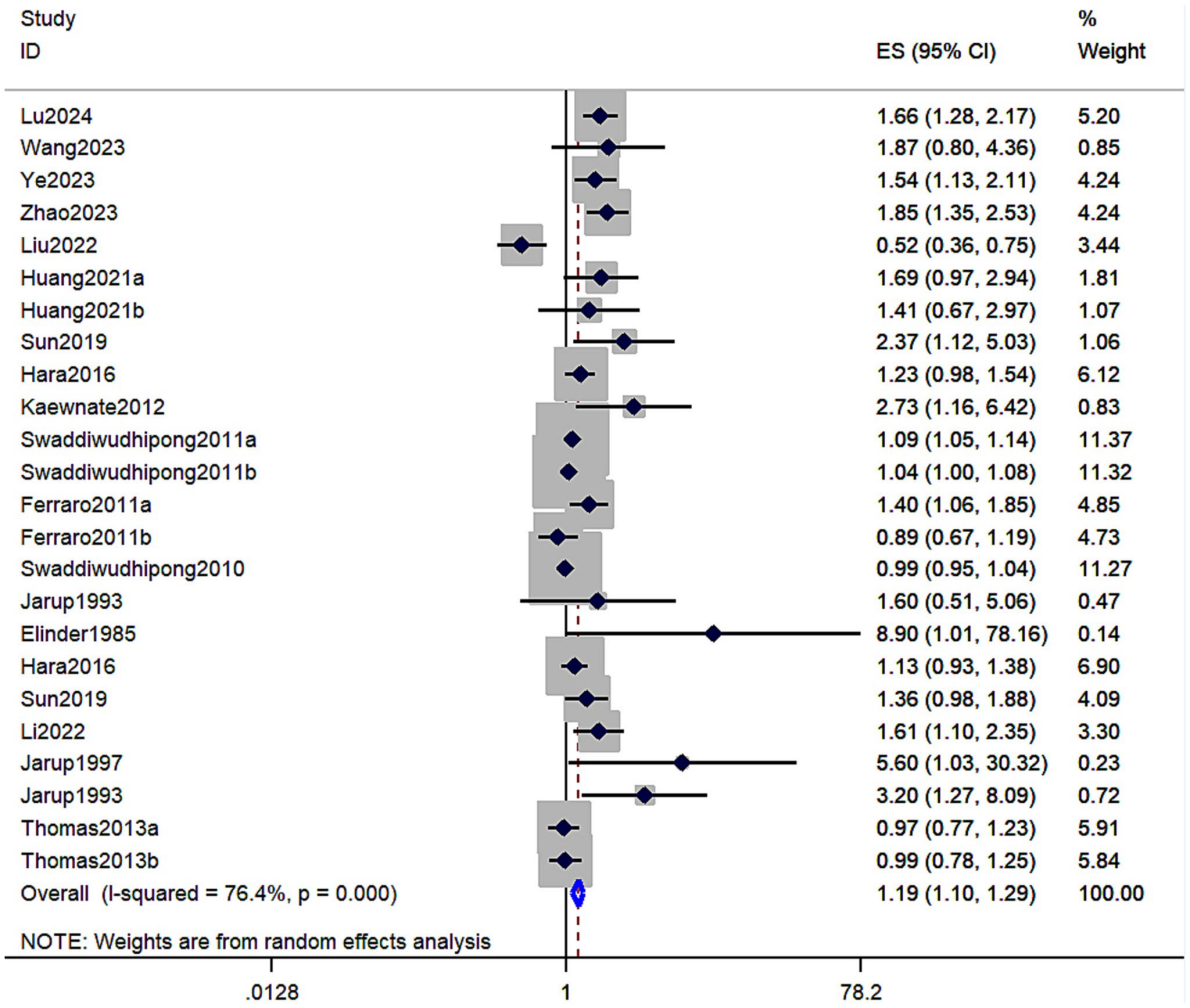
Forest plot showing the association between cadmium exposure and the risk of kidney stones. The results showed that cadmium exposure was associated with an increased risk of kidney stones (RR = 1.19; 95% CI: 1.10–1.29).

A total of 17 studies involving 159,011 individuals evaluated the association between cadmium exposure and the risk of kidney stones. The results showed that cadmium exposure was associated with an increased risk of kidney stones (RR = 1.19; 95% CI: 1.10–1.29, Figure 2). Significant heterogeneity was observed among the evaluated studies (*I*^2^ = 76.4%, *p* < 0.001). Table 2 shows the results of the subgroup analyses performed to evaluate any potential effects of sex, study design, region, and exposure assessment method on these associations.

**Table 2:** Subgroup analyses of the studies included in the meta-analysis.

Subgroup	Number of studies	Pooled RR (95% CI)	*I*^2^ statistics (%)	*P*-value for the heterogeneity Q test
Urinary cadmium levels	14	1.19 (1.08, 1.30)	80.00	<0.001
Female	6	1.36 (0.94, 1.98)	71.90	0.002
Male	7	1.01 (0.77, 1.33)	83.50	<0.001
Mixed^*^	10	1.66 (1.27, 2.18)	84.70	<0.001
Cadmium-contaminated areas	7	1.08 (1.00, 1.15)	67.80	0.002
Non-contaminated areas	6	1.33 (0.97, 1.82)	83.90	<0.001
Asian	5	1.03 (0.94, 1.13)	81.60	<0.001
Non-Asian	9	1.47 (1.21, 1.77)	57.10	0.013
Cross-sectional studies	12	1.16 (1.06, 1.27)	81.90	<0.001
Case–control studies	2	1.78 (1.20, 2.64)	0.00	0.504
Blood cadmium levels	5	1.49 (1.10, 2.02)	59.00	0.045
Female	1	1.57 (1.05, 2.34)	–	–
Male	1	1.23 (0.77, 1.97)	–	–
Mixed^*^	5	1.49 (1.10, 2.02)	59.00	0.045
Cadmium-contaminated areas	4	1.69 (1.05, 2.71)	68.90	0.022
Non-contaminated areas	1	1.36 (0.98, 1.88)	–	–
Asian	1	1.61 (1.10, 2.35)	–	–
Non-Asian	4	1.51 (1.01, 2.25)	63.60	0.041
Cross-sectional studies	4	1.51 (1.01, 2.25)	63.60	0.041
Case–control studies	1	1.61 (1.10, 2.35)	–	–
Dietary levels	1	0.98 (0.83, 1.16)	–	–
Cohort	1	0.98 (0.83, 1.16)	–	–

*The study population included both male and female individuals; RR, Relative risk.

**Figure 3:**
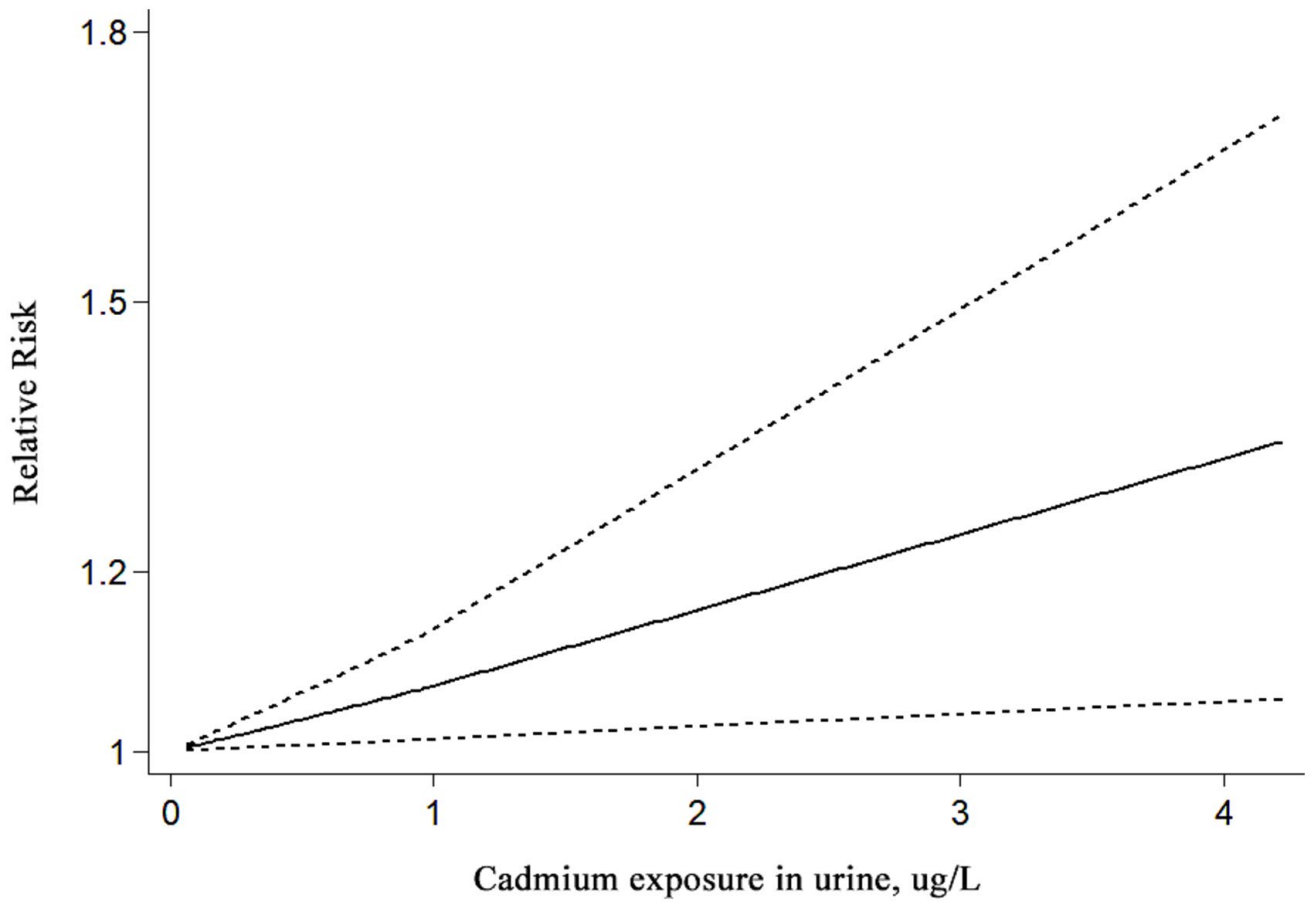
Linear dose–response association between urinary cadmium levels and the risk of kidney stones. Analyses were conducted using a fixed effects model. A significant increase in the risk of kidney stones was observed for each additional 1 μg/L of cadmium in urine.

In addition, 14 studies involving 89,630 individuals in total evaluated the association between urinary cadmium exposure levels and the risk of kidney stones. The results showed that urinary cadmium exposure was associated with an increased risk of kidney stones (RR = 1.19; 95% CI: 1.08–1.30; *p* < 0.001; Table 2). A high degree of heterogeneity was observed among the evaluated studies (*I*^2^ = 80.00%, *p* < 0.001). The results of the subgroup analyses based on sex showed that cadmium exposure was associated with an increased risk of kidney stones in mixed-sex populations (RR = 1.66; 95% CI: 1.27–2.18, *I*^2^ = 84.70%, *p* < 0.001), compared to women (RR = 1.36; 95% CI: 0.94 to 1.98, *I*^2^ = 71.90%, *p* = 0.002) and men (RR = 1.01; 95% CI: 0.77–1.33, *I*^2^ = 83.50%, *p* < 0.001) (Table 2). Moreover, a statistically significant increased association was observed in cadmium-contaminated areas (RR = 1.08; 95% CI: 1.00–1.15, *I*^2^ = 67.8%, *p* = 0.002), but no association between higher cadmium exposure and the risk of kidney stones was observed in non-contaminated areas (RR = 1.33; 95% CI: 0.97–1.82, *I*^2^ = 83.90%, *p* < 0.001) (Table 2). The subgroup analyses based on study design showed that cadmium exposure was associated with an increased risk of kidney stones in both case–control studies (RR = 1.78; 95% CI: 1.20–2.64, *I*^2^ = 0.00%, *p* = 0.504) and cross-section studies (RR = 1.16; 95% CI: 1.06–1.27, *I*^2^ = 81.90%, *p* < 0.001) (Table 2). The results of the subgroup analyses by region showed that cadmium exposure was associated with an increased risk of kidney stones in non-Asian populations (RR = 1.47; 95% CI: 1.21–1.77, *I*^2^ = 57.10%, *p* < 0.013), whereas no significant association was observed in Asian populations (RR = 1.03; 95% CI: 0.94–1.13, *I*^2^ = 58%, *p* = 0.015) (Table 2). Furthermore, based on the three studies included in the linear dose–response meta-analysis, a significant association was observed between urinary cadmium exposure levels and the risk of kidney stones. Each additional 1 μg/L increase in urinary cadmium was associated with a 7% higher risk of kidney stones (RR = 1.07; 95% CI 1.01–1.13; *I*^2^ = 26.4%; three studies; 1,769 cases; range of cadmium level = 0.08–4.225ug/L; Figure 3).

A total of five studies involving 4,425 individuals in total evaluated the association between blood cadmium exposure levels and the risk of kidney stones. A significant association was found between blood cadmium exposure levels and the risk of kidney stones (RR = 1.49; 95%CI: 1.10–2.02, *I*^2^ = 59%, *p* = 0.045, Table 2) The results of the subgroup analyses by sex showed that cadmium exposure was associated with an increased risk of kidney stone disease in female individuals (RR = 1.57; 95% CI: 1.05–2.34) and mixed-sex populations (RR = 1.49; 95% CI: 1.10–2.02, *I*^2^ = 59%, *p* = 0.045) compared to male individuals (RR = 1.23; 95% CI: 0.77–1.97) (Table 2). In the subgroup analyses using a random effects model, there were significant associations between subgroups based on study design and region (Table 2). Moreover, there was a statistically significant increased risk in cadmium-contaminated areas (RR = 1.69; 95% CI: 1.05–2.71, *I*^2^ = 68.9%, *p* = 0.022), while no association was observed between higher cadmium exposure and the risk of kidney stones in non-contaminated areas (RR = 1.36; 95% CI: 0.98–1.88) (Table 2).

One cohort study examined the association between higher cadmium exposure in dietary sources and the risk of kidney stones, and the results showed no significant association (RR = 0.98; 95% CI: 0.83–1.16) (Table 2).

### Sensitivity analysis and publication bias

**Figure 4:**
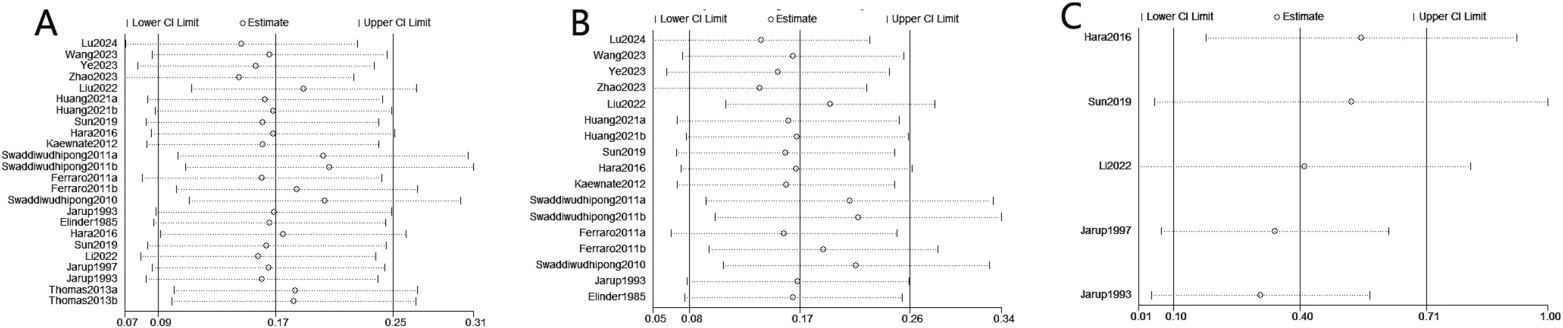
Sensitivity analysis diagrams for the studies assessing the association between cadmium exposure and the risk of kidney stones. **(A)** Cadmium exposure and the risk of kidney stones; **(B)** Urinary cadmium exposure levels and the risk of kidney stones; **(C)** Blood cadmium levels and the risk of kidney stones.

**Figure 5:**
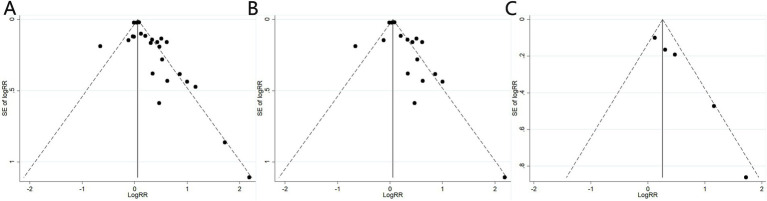
Funnel plots of the studies assessing the association between cadmium exposure and the risk of kidney stones. **(A)** Cadmium exposure and the risk of kidney stones; **(B)** Urinary cadmium exposure levels and the risk of kidney stones; **(C)** Blood cadmium levels and the risk of kidney stones.

A sensitivity analysis was conducted to assess the risk of kidney stones by excluding individual studies one at a time, and the results showed that no individual study influenced the overall RRs (Figure 4), indicating that the results of this meta-analysis are relatively stable. Publication bias was observed in the results based on the Egger’s test and funnel plots (Table 3; Figure 5).

**Table 3:** Publication bias test for the association between cadmium exposure and the risk of kidney stones.

Exposure		Egger’s test		Begg’s test
	Coefficient	*P*	95% CI	
Cadmium	1.441	0.003	0.540–2.343	0.130
Urinary cadmium levels	1.459	0.025	0.208–2.709	0.773
Blood cadmium levels	2.532	0.005	1.447–3.617	0.086

## Discussion

A total of 17 studies involving 159,011 participants met the inclusion criteria and were eventually included in our systematic review and meta-analysis. Overall, our results indicated that the risk of kidney stones was increased in individuals with higher cadmium exposure. Importantly, we observed an increased risk of kidney stones in individuals living in cadmium-contaminated areas, while no such risk was found in non-contaminated areas. Moreover, the dose–response meta-analysis indicated that an increase of 1ug/L in urinary cadmium was associated with a 7% rise in the risk of developing kidney stones. These findings are of great significance, as they highlight the association between cadmium exposure and the risk of kidney stones and may help in preventing the formation of kidney stones by minimizing exposure to cadmium, especially in cadmium-contaminated areas.

The heavy metal cadmium is one of the most toxic industrial and environmental pollutants, which poses a severe threat to human health (10, 11). Cadmium can enter the body through air, water, soil, and food, and it largely accumulates in the kidneys, liver, bones, and other organs, causing irreversible damage to the target organs (18, 40). Higher cadmium exposure has also been considered a possible risk factor for kidney stones. Similar to our findings, higher cadmium exposure has previously been associated with the risk of kidney stones in several studies utilizing data from the National Health and Nutrition Examination Survey (NHANES) (24, 36, 37). Kaewnate et al. also observed an association between elevated levels of urinary cadmium and urinary stones among 1,085 study residents from 13 cadmium-contaminated villages in Thailand (19). In their results, elevated levels of urinary cadmium appeared to increase the risk of urinary stones, with an adjusted odds ratio (OR) of 2.73 and a 95% confidence interval (CI) of 1.16–6.42, after adjusting for other co-variables (19). A case–control study conducted by Li et al. showed that the ratio of plasma cadmium to kidney stones in the highest quartile was 1.606 (95% CI, 1.100–2.344) compared to the lowest quartile in rural areas of Guangxi, China (32). However, several studies found no association between higher cadmium exposure and the risk of kidney stones. Hara et al. observed that higher levels of blood cadmium and urinary cadmium were not associated with an increased risk of kidney stones (30). Liu et al. reported that higher blood cadmium exposure was not associated with the risk of kidney stones in the Qiandongnan Prefecture, China (25). This difference between studies may be due to the study design, sample size, nationalities, or study regions. Thus, more high-quality studies are needed to further assess the associations.

A meta-analysis conducted by Guo et al., which included six studies with a total of 88,045 participants, found that higher cadmium exposure was significantly associated with an increased risk of urolithiasis, with a 1.32-fold increase in risk (41). Their study focused exclusively on cadmium levels in urine and dietary intake. Compared to the previous meta-analysis, we extended this investigation by including blood cadmium levels as an additional factor. Consequently, we included more studies with a larger sample size and performed multiple subgroup analyses to assess heterogeneity and publication bias. According to the subgroup analysis, urinary and blood cadmium levels were associated with the risk of kidney stones, while dietary cadmium exposure was not significantly associated with the risk of kidney stones. A low exposure dose and limited number of studies may explain the lack of association between dietary cadmium exposure and the risk of kidney stones. In addition, a linear dose–response meta-analysis was conducted, which showed a significant association between cadmium exposure in urine and the risk of kidney stones, with an increased risk observed for every additional 1 μg/L of cadmium in urine. In the subgroup meta-analyses based on study design, we found that higher cadmium exposure was associated with an increased risk of kidney stones in both case–control and cross-sectional studies. We also observed a higher risk of kidney stone disease in individuals living in cadmium-contaminated areas but not in those from non-contaminated areas. One possible explanation for the lack of association in non-contaminated areas is that exposure levels were not high enough to significantly affect the urinary composition. The results of the subgroup analyses based on sex showed that urinary cadmium exposure was associated with an increased risk of kidney stones in mixed-sex populations compared to women and men. Similarly, blood cadmium exposure was not associated with an increased risk of kidney stones in men. The subgroup analyses based on ethnicity showed that urinary cadmium exposure was not associated with an increased risk of kidney stone disease in Asian populations compared to non-Asian populations. This lack of association is likely due to the limited number of studies included in the meta-analysis. Cadmium exposure may lead to renal stone formation, but the exact mechanism is not clear. Cadmium has a long half-life, and once it enters the body, it accumulates irreversibly in the kidneys (42, 43). Cadmium accumulation in the kidneys leads to a higher calcium excretion rate, which can raise the likelihood of developing kidney stones (12, 44). Cadmium can recombine with metallothionein produced by renal tubular epithelial cells, causing significant damage to kidney tubular cells and impairing their reabsorption function (45, 46). Cellular injury in renal tubular epithelial cells induces crystal nucleation and aggregation, and this may result in ineffective crystallization modulators and localized areas of supersaturation in the interstitial space (47). Kidney stones may form as a result of this process. In addition, sexual hormones may play an important role in the development of nephrolithiasis (47, 48). As an endocrine-disrupting chemical, cadmium may contribute to the formation of renal stones by disrupting endocrine functions (49).

For the purpose of reporting our observations, we conducted a comprehensive literature search following the PRISMA guidelines. Our study included 17 studies involving 159,011 participants to evaluate the relationship between cadmium exposure and the risk of kidney stones. The large sample size is an important strength of this study. However, there were several limitations. First, the studies included in the meta-analysis showed a high level of heterogeneity, which persisted even after extensive sensitivity analysis and several subgroup analyses. Second, there were too few studies to draw a definitive conclusion about the risk of kidney stones in people with dietary cadmium exposure. Finally, due to limited data, we could not assess the dose–response relationship between blood cadmium levels and the risk of kidney stones. Similarly, we were unable to assess the effects of age, smoking habits, ethnicity, and study quality on the risk of kidney stones associated with cadmium exposure. Therefore, more prospective cohort studies that evaluate the incidence of kidney stones in relation to cadmium exposure are needed.

The results of this meta-analysis strengthen the evidence that higher cadmium exposure is a risk factor for kidney stones. Further detailed research is needed to better understand the mechanisms underlying these associations. Efforts to reduce cadmium exposure in the population may help reduce the individual, economic, and societal burdens of kidney stones.

## Data Availability

The original contributions presented in the study are included in the article/supplementary material, further inquiries can be directed to the corresponding author.
